# The effect of temperature on asexual reproduction in *Hydra vulgaris*

**DOI:** 10.17912/micropub.biology.001508

**Published:** 2025-04-21

**Authors:** Jameelah Destry, Kelso Cochran, Aide Macias-Muñoz

**Affiliations:** 1 Biological Sciences, University of Maryland, Baltimore, Baltimore, Maryland, United States; 2 Ecology and Evolutionary Biology, University of California, Santa Cruz, Santa Cruz, California, United States; 3 Geography and Environmental Systems, University of Maryland, Baltimore, Baltimore, Maryland, United States

## Abstract

*Hydra vulgaris* is a model cnidarian used for interdisciplinary studies in biology, yet its reproductive responses to environmental changes remain underexplored. This study examined how temperature affects asexual reproduction (budding) rates and population growth in *H. vulgaris* . We placed *Hydra* in two thermal environments, 15°C and 25°C, to compare differences in population growth, number of budding polyps, number of buds, and budding rates under ‘cold’ and ‘hot’ conditions. Our findings indicate that *Hydra* populations exhibit increased growth through asexual budding at higher temperatures.

**Figure 1. Asexual reproduction in Hydra under different temperatures:**
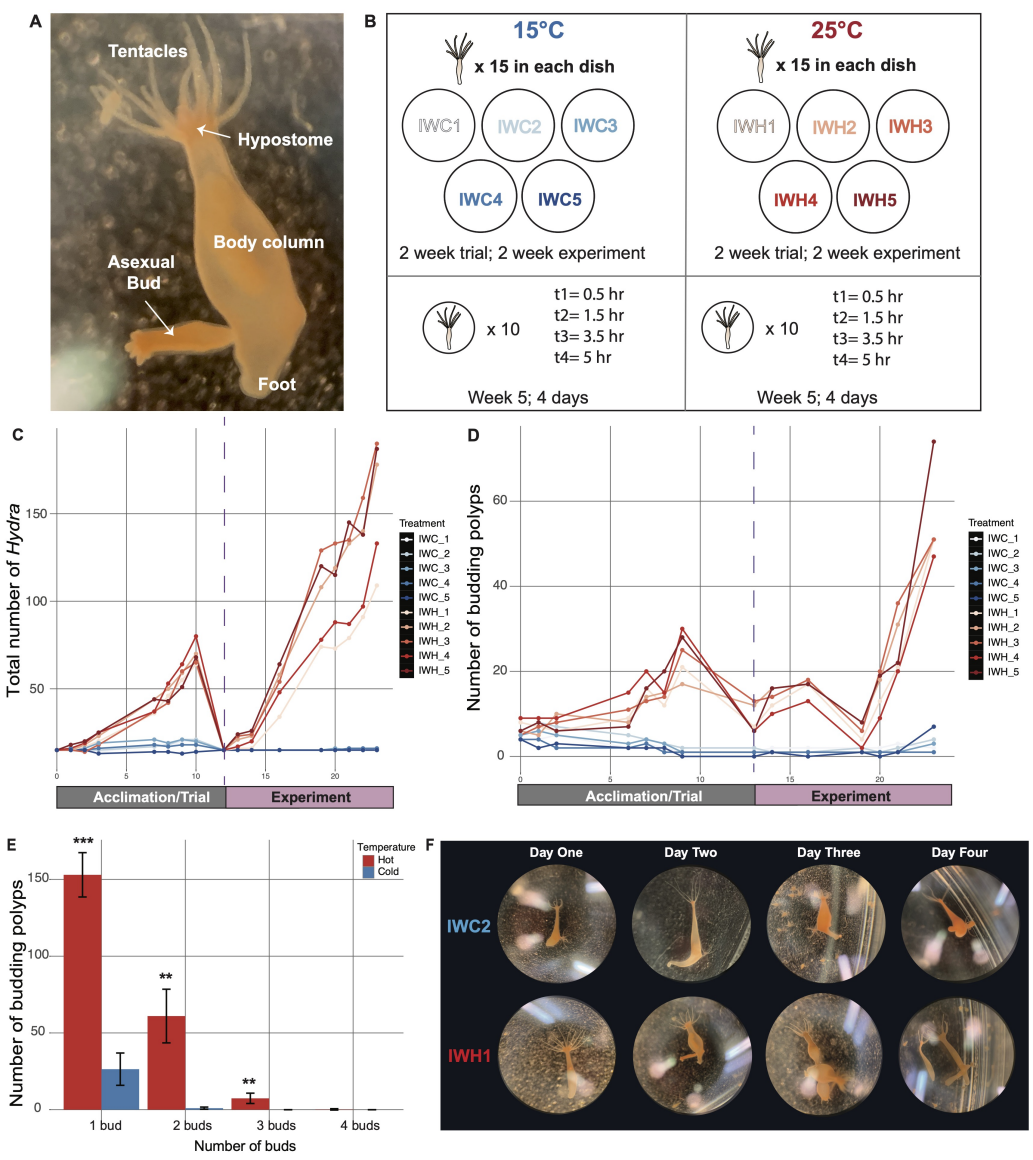
A) Photograph of a *Hydra* polyp with tentacles, hypostome (mouth opening), body column, foot, and developing asexual bud labeled. B) Schematic of experimental set-up. IWC is inverse watermelon cold population and IWH are inverse watermelon hot populations; 5 replicates in each treatment. C) Total number of *Hydra* in each dish during the acclimation/trial period and experimental period; both beginning with 15 individuals in each dish. Dashed line indicates the start of the experimental period. D) Total number of budding *Hydra* in each dish during the acclimation/trial period and experimental period. E) Average number of budding *Hydra* with 1, 2, 3 or 4 buds present per polyp across dishes throughout the experiment. The error bars display the standard deviation; *** p<0.001, ** p<0.01. F) Photographs of representative budding *Hydra* in the cold and hot room.

## Description

*H. vulgaris* are freshwater polyps that belong to the phylum Cnidaria, which encompasses jellyfish, sea anemones, and corals. *H. vulgaris* have been used as a model organism in biological studies exploring genetics, stem cells, neurogenesis, aging, stress responses, development, and regeneration (Galliot 2012). Several laboratories have incorporated *H. vulgaris* into their research due to their ease of rearing. *H. vulgaris* have a simple body plan consisting of two germ layers that form its body column, foot, hypostome (mouth), tentacles, and are approximately 1 mm in length and 0.3 mm wide ( Fig. 1A ). Moreover, *H. vulgaris* are also capable of reproducing both sexually and asexually by budding. Under favorable conditions, a bud develops in the lower section of the *H. vulgaris* body. The bud proceeds through 10 stages of growth and tentacle formation, then detaches from the parent polyp (Otto & Campbell 1977). In this study, we investigated how *H. vulgaris* responds to two thermal environments. Specifically, we characterized: 1) changes in population size, 2) number of budding individuals, 3) differences in number of buds produced, and 4) rate of budding under ‘hot’ and ‘cold’ conditions. To do this, we placed *H. vulgaris* at 15°C (cold) and 25°C (hot) for 5 weeks; the first 2 weeks were used as an acclimation and trial period, followed by 2 weeks of an experimental period. The fifth week was dedicated to individual *H. vulgaris* polyp observations (Fig 1B).

Previous studies have investigated the effects of temperature and diet on body size and population growth rate in some species of *Hydra* . One study found that *Hydra oligactis* maintained at 13°C, 20°C, and 27°C, showed a negative correlation between temperature and polyp size (Hecker & Slobodkin). At higher temperatures, individuals are smaller with fewer cells than the larger polyps with more cells at colder temperatures (Hecker & Slobodkin). In another species, *Hydra littoralis* acclimated to 5°C, 10°C, 15°C and 21° C, a similar correlation between temperature and polyp size were observed (Park & Ortmeyer 1972). *Hydra littoralis* acclimated to lower temperatures were larger than polyps acclimated to higher temperatures (Park & Ortmeyer 1972). Budding rates were also found to increase with temperature. In addition, sudden shifts in temperature led to initial amplified or diminished budding rates compared to acclimated polyps (Park & Ortmeyer 1972). Moreover, a study comparing *Hydra pseudoligactis* , *Hydra viridissima* , and *Hydra littoralis* concluded that temperature and feeding frequency have a direct effect on budding, but the effect was variable among species (Stiven 1965). With the rise of *H. vulagris* as a model organism for interdisciplinary research, we sought to characterize the effects of temperature on its asexual reproduction.

Our study found that *H. vulgaris* in the hot temperature (25°C) were undergoing asexual reproduction more frequently than in the cold (15°C) (p < 2e-16; generalized linear model, population size ~ temperature * day + replicate) (Fig 1C). During the trial period, populations at 25°C rose to an average of 69 polyps while populations at 15°C averaged 19 polyps. During the experimental week, populations at 25°C rose to an average of 159 polyps while populations at 15°C stayed at an average of 15 polyps per dish ( Figure 1C ). In terms of budding polyps, *H. vulgaris* populations at 25°C had more budding individuals than those kept at 15°C (p < 2e-16: generalized linear model, number of budding individuals ~ temperature + day) (Fig 1D). By the end of experimental weeks, an average of 45 polyps were budding at 25°C compared to 4 at 15°C. Interestingly, we noticed decreases in budding individuals occurred after weekends when *H. vulgaris* were not fed ( Figure 1D ). In terms of the number of buds per individual, most budding polyps at both temperatures had 1 bud (average 158 polyps at 25°C and 28 polyps at 15°C) (Fig 1E).

After the initial experiment was concluded, we transferred 10 polyps into individual petri dishes to track stages of bud development over the course of 4 days (Fig 1B bottom). In general, we found that buds developed and separated within 2 days at 25°C. Meanwhile, at 15°C, buds had not separated (or budded off) after 4 days (representative *Hydra* shown in Fig 1F). By the end of the experiment, a polyp at 25°C was able to produce two new buds that detached by the end of 4 days. Conversely, a polyp at 15°C was able to produce two new buds but none grew into a full detached adult *Hydra* by the end of 4 days.

Overall, our results show that *H. vulgaris* undergo asexual reproduction by budding more readily in the warmer temperature. A limitation of the study is that it was carried out in one summer. It is possible that the acclimation period between the ‘hot’ and ‘cold’ temperatures may be different, and longer than 2 weeks for the cold temperature. *H. vulgaris* in the ‘hot’ conditions were consistently producing new buds within the first 2 weeks of the experiment but polyps in the ‘cold’ condition were still in the process of budding off at the end of our 4-week experiment. It would be interesting to investigate how long it takes the *H. vulgaris* to acclimate to hot and cold temperatures in general. Another limitation is that we did not have an accurate reading of the laboratory room temperature before the experiment was conducted, otherwise we would have tried to mirror temperature changes. A future direction includes identifying some of the potential cascading effects of this type of growth in their natural environments. Global climate change is shifting temperatures and climates in unprecedented ways resulting in extreme temperatures and the expansion or shrinking of different animal habitats due to these changes (Seehausen et al. 2008). An ecosystem containing an organism whose reproduction is influenced by temperature could be impacted by sudden population changes. Anticipated warmer temperatures due to climate change could lead to either positive or negative effects on their food webs. Investigating the temperature changes that *H. vulgaris* populations experience in the wild and their responses would be another interesting research direction.

## Methods

*Hydra Care*

In the laboratory setting, *Hydra vulgaris* are kept in a pyrex at room temperature (~22°C) and fed 3-4 times per week. For this experiment, *H. vulgaris* (strain: inverse watermelon) were kept in petri dishes (diameter 150mm and depth 15mm) in *Hydra* medium. *Hydra* were placed in one of two controlled environment rooms (CERs). One room was set to 25°C with a 14/10 daylight cycle (hot room) and the other was set to 15°C with a 10/14 light/dark cycle (cold room). Temperature ranges were chosen based on previous studies (Schroeder & Callaghan; Kaliszewicz & Lipińska 2013). The difference in *Hydra* at room temperature to the ‘cold’ and ‘hot’ CER conditions were -7 and +3, respectively. *Hydra* were fed San Francisco Bay strain *Artemia salina* daily Monday through Friday. *Hydr* a were allowed to feed for approximately 2 hours before cleaning. Cleaning consisted of removing all artemia and adding fresh *Hydra* medium (see https://openhydra.org/wp-content/uploads/2019/09/Hydra_Culturing_Protocol.pdf). Each CER had its own clean *Hydra* medium acclimated to the temperature of the room.

*Observations of Asexual Reproduction and Number of Budding Individuals*

5 petri dishes with 15 hydra polyps each were placed in each of the cold and hot CERs. Specimens were transferred from room temperature to experimental conditions. The total length of this experiment was 4 weeks. The first two weeks were a trial period during which we noted acclimation took ~1 week. Observations of the total number of polyps, the number of individuals undergoing asexual reproductions, and how many buds each *Hydra* bore were recorded for five consecutive days. Date, time, temperature and humidity of CERs were recorded during daily animal care. After the trial period, we reduced populations down to 15 polyps again and began the experimental period.

*Observation of Budding Rate*

To detect budding rates at the individual level, we tracked bud development in 10 individual polyps. We took 10 *Hydra* polyps from each CER and placed each in a small petri dish (circumference 30mm and depth 15mm). The *Hydra* were fed in the morning (T0). Observations and photographs were taken every day at: 0.5 hrs, 1.5 hrs, 3.5 hrs, 5.5 hrs (Day 1), and 5.0 hrs (Days 2-4) from T0. Photographs were taken using a 12MP wide Iphone XR camera and a Fisherbrand Stereo Zoom Microscope (Catalog #03000020). The experiment lasted four days.
